# Two Novel EHEC/EAEC Hybrid Strains Isolated from Human Infections

**DOI:** 10.1371/journal.pone.0095379

**Published:** 2014-04-21

**Authors:** Rita Prager, Christina Lang, Philipp Aurass, Angelika Fruth, Erhard Tietze, Antje Flieger

**Affiliations:** Divison of Enteropathogenic Bacteria and *Legionella*, National Reference Centre for *Salmonella* and other Bacterial Enteric Pathogens, Robert Koch-Institut, Wernigerode, Germany; University of Osnabrueck, Germany

## Abstract

The so far highest number of life-threatening hemolytic uremic syndrome was associated with a food-borne outbreak in 2011 in Germany which was caused by an enterohemorrhagic *Escherichia coli* (EHEC) of the rare serotype O104:H4. Most importantly, the outbreak strain harbored genes characteristic of both EHEC and enteroaggregative *E. coli* (EAEC). Such strains have been described seldom but due to the combination of virulence genes show a high pathogenicity potential. To evaluate the importance of EHEC/EAEC hybrid strains in human disease, we analyzed the EHEC strain collection of the German National Reference Centre for *Salmonella* and other Bacterial Enteric Pathogens (NRC). After exclusion of O104:H4 EHEC/EAEC strains, out of about 2400 EHEC strains sent to NRC between 2008 and 2012, two strains exhibited both EHEC and EAEC marker genes, specifically were *stx2* and *aatA* positive. Like the 2011 outbreak strain, one of the novel EHEC/EAEC harbored the Shiga toxin gene type *stx2a.* The strain was isolated from a patient with bloody diarrhea in 2010, was serotyped as O59:H^−^, belonged to MLST ST1136, and exhibited genes for type IV aggregative adherence fimbriae (AAF). The second strain was isolated from a patient with diarrhea in 2012, harbored *stx2b*, was typed as Orough:H^−^, and belonged to MLST ST26. Although the strain conferred the aggregative adherence phenotype, no known AAF genes corresponding to fimbrial types I to V were detected. In summary, EHEC/EAEC hybrid strains are currently rarely isolated from human disease cases in Germany and two novel EHEC/EAEC of rare serovars/MLST sequence types were characterized.

## Introduction

*Escherichia coli* bacteria on the one hand belong to the normal flora of the human intestine but on the other hand may cause disease. Pathogenic *E. coli* variants harbor specific genes encoding virulence determinants [1]. For example, enterohemorrhagic *E. coli* (EHEC) are an intestinal pathovar that cause about 1,000 to 1,500 cases of diarrhea or bloody diarrhea in Germany per year and about 70 cases of the severe pathology hemolytic uremic syndrome (HUS) [2]. For the large outbreak of bloody diarrhea/HUS in Germany in 2011, 53 deaths, 833 HUS cases, and about 3,000 cases of gastroenteritis were recorded [3]. Although Shiga toxin-positive strains inducing HUS in many cases belong to the serovar O157:H7/H^−^ and others such as O26:H11/H^−^, O103:H2/H^−^, O111:H8/H^−^, O145:H28/H^−^, the outbreak was caused by an *E. coli* strain of the rare serotype O104:H4 characterized by the presence of EHEC pathovar markers as well as genes of another pathovar, enteroaggreagative *E. coli* (EAEC). So called mixed *E. coli* pathovars or hybrid strains have been seldom described and show a high virulence potential [4]–[16].

Important virulence determinants of classical EHEC are the Shiga toxin Stx, a type III-protein secretion system coded on a pathogenicity island, the locus of enterocyte effacement (LEE), and the EHEC toxin Ehx encoded by the gene *ehxA*. Stx is responsible for severe pathologies as observed for HUS, LEE induces intimate attachment of the bacteria to the intestinal epithelia, and Ehx is a pore-forming toxin [1], [17]. An Stx gene, especially for the more toxic type Stx2, but not LEE- or Ehx-related genes were found in the 2011 EHEC/EAEC outbreak strain [4], [5], [7], [9], [12], [13].

Classical EAEC do not possess *stx* genes but encode aggregative adherence fimbriae (AAF) on the virulence plasmid pAA which cause the characteristic stacked brick-like adherence of the bacteria to host cells [18], [19]. So far, five different types of such fimbriae are known and the 2011 outbreak strain coded type I aggregative fimbriae [4], [6], [7], [20]–[23]. The virulence plasmid of EAEC not only encodes the aggregative adherence fimbrial subunit genes but in many cases also an ABC transporter complex Aat involved in the export of the antiaggregation protein dispersin (Aap), and the AraC-like regulator AggR which drives their expression but also expression of chromosomally localized genes [24]–[27]. EAEC have first been found associated with persistent diarrhea mainly in children in developing countries but are increasingly recognized as a cause of diarrhea in industrial countries [25], [28].

Analysis of the 2011 outbreak strain uncovered that its core genome is related to an earlier described classical EAEC of serovar O104:H4 designated 55989 and suggested additional acquisition of some EHEC features [5], [9], [12], [13], [16], [29], [30]. Further, genome sequencing determined the relation of the outbreak strain to another O104:H4 EHEC/EAEC isolated from HUS cases in 2001. It was hypothesized that the two EHEC/EAEC strains and the EAEC strain of the same serovar share a common ancestor [9]. Differences between the three strains which all belong to the MLST sequence type ST678 were further noted in the antibiotic resistance profile, the plasmid profile, and the macrorestriction/pulsed-field electrophoresis (PFGE) pattern [4], [5], [7], [9].

To evaluate the significance of EHEC/EAEC hybrid strains in human disease, we here analyzed strains from the EHEC strain collection of the German National Reference Centre for *Salmonella* and other Bacterial Enteric Pathogens (NRC). In the repository of EHEC strains collected between 2008 and 2012, two strains exhibiting both EHEC and EAEC marker genes were found. These were further analyzed in our study and compared to the 2011 outbreak strain, classical EAEC and EHEC.

## Materials and Methods

### Strains

The strains used in the study are listed in Table 1. Strains were grown on nutrient agar (Oxoid GmbH, Germany) or in tryptic soy broth (TSB) (BD-BBL, Germany), if not stated otherwise. Testing of hemolytic activity was performed on enterohemolysin agar (Sifin GmbH, Germany).

**Table 1:** Characteristics of EHEC/EAEC, EAEC, and EHEC strains analyzed in the study.

Pathovar/notes	Isolatenumber	Date/yearof isolation	Symptoms	Serovar	Phylogroup	MLSTST	*aatA*	AAFtype	*stx1*	*stx2*	*eaeA*	*ehxA*	Plasmids[MDa]	Antibioticresistance
**Novel EHEC/EAEC,** **this study**	**10-06235**	**12.11.2010**	**Bd**	**O59:H^−^ (** ***fliC_H19_*** **)**	**B1**	**1136**	**+**	***hdaA/C*** ** (type IV)**	**−**	**+**	**−**	**−**	**50, 60** * **, 70**	**SMZ, STR, SXT**
**Novel EHEC/EAEC,** **this study**	**12-05829**	**5.12.2012**	**diarrhea**	**Orough:H^−^ (** ***fliC_H8_*** **)**	**B1**	**26**	**+**	**unknown**	**−**	**+**	**−**	**−**	**40, 75** * **, 80**	**susceptible**
EHEC/EAECHUSEC 041Germany [48]	01**-**09591	2001	Bd, HUS	O104:H4	B1	678	+	*agg3A/C* (type III)	**−**	+	**−**	**−**	3,4; 5,5; 46*; 60;	AMP, MEZ, NAL, SMZ, STR
EHEC/EAECoutbreak 2011Germany [7]	11**-**02027	19.5.2011	Bd, Hc, HUS	O104:H4	B1	678	+	*aggA/C* (type I)	**−**	+	**−**	**−**	55*, 60	AMP, CAZ, CTM, CTX, MEZ, MSU, NAL, OTE, SMZ, STR, SXT
EAEC,this study	05**-**08045	3.11.2005	diarrhea	O106:H^−^ (*fliC_H18_*)	D1	414	+	*aafA/C* (type I)	**−**	**−**	**−**	**−**	80	NAL
EAEC,this study	06**-**06057	31.8.2006	unknown	O92:H33	A1	34	+	*aggA/C* (type I)	**−**	**−**	**−**	**−**	60	AMP, MEZ, NAL, OTE, SMZ, SXT
EAEC,this study	10**-**03550	21.7.2010	unknown	O111:H21	B1	40	+	*aaf5A/agg3C* (type V)	**−**	**−**	**−**	**−**	60	AMP, CTM
EAEC,this study	10**-**06623	28.10.2010	unknown	O173:H6	B1	278	+	*hdaA/C* (type IV)	**−**	**−**	**−**	**−**	98	susceptible
EAEC,this study	11**-**08343	28.10.2011	Bd	O59:H^−^ (*fliC_H19_*)	B1	1136	+	*hdaA/C* (type IV)	**−**	**−**	**−**	**−**	55, 60*	susceptible
EHECReference strain EDL933 [62], [63]	CDC EDL933	1982	RawHamburgermeat	O157: H7	B1	11	**−**	n.a.	+	+	+	+	61	n.d.

MLST sequence types were assigned or cited according to the MLST database at the University College Cork, Ireland [46]. Abbreviations: Bd = bloody diarrhea, Hc = hemorrhagic colitis, n.d. = not determined, n.a. = not applicable, AMP = ampicillin, CTM = Cefotiam, MEZ = mezlocillin, MSU = mezlocillin/sulbactam, OTE = oxytetracyclin, SMZ = sulfamethoxazol, STR = streptomycin, SXT = trimethoprim/sulfamethoxazol, CAZ = ceftazidim, CTX = cefotaxim, and NAL = nalidixic acid.

*-pAA plasmid.

### Triplex PCR for Detection of *stx1*, *stx2*, and *aatA*

For simultaneous detection of *stx1/2* (EHEC marker) and *aatA* (EAEC marker), colony PCR was performed. *stx1/2* primer sequences have been used from Cebula et al. (LP30: CAG TTA ATG TGG TGG CGA AGG, LP31: CAC CAG ACA ATG TAA CCG CTG and LP43 ATC CTA TTC CCG GGA GTT TAC G, LP44: GCG TCA TCG TAT ACA CAG GAG C) [31]. *aatA* primers designed in this study are directed to conserved regions within the gene (aatA_fw: TCG GCT TAT GAA GCA AAA ATG; aatA_rv: GAT AAC GTC GTC TTG TCC ATT C). Each reaction contained 2.5 µl of 10×PCR buffer (NEB), 1 unit of Taq DNA polymerase (NEB), 5 pmol of each forward and reverse primers, 200 µM of each deoxynucleoside triphosphate (Bioline) and distilled water up to a total reaction volume of 15 µl. A small amount of a single bacterial colony resuspended in 10 µl distilled water and heated for 10 min at 95°C was used as DNA template. DNA amplification was carried out in a PCR thermal cycler. The following PCR conditions were used: 94°C for 5 min, followed by 30 cycles of 30 seconds at 94°C, 1 min at 55°C, and 1 min at 72°C with a final extension of 5 min at 72°C. Separate PCR analysis for *stx1/2* or *aatA* was performed at comparable conditions but as a duplex or single assay, respectively.

### PCR Analysis for Differentiation of AAF Fimbriae

The AAF variants AAF/I, AAF/II, AAF/III and AAF/IV (or Hda) were determined by PCR amplification of the corresponding subunits and usher genes (*aggA*, *aggC*, *aafA*, *aafC*, *agg3A*, *agg3C*, *hdaA*, *hdaC*) as described previously [20], [21], [23], [32]–[34]. A PCR assay was developed for detection of the AAF/VA subunit gene. The primers (aaf5_fw: TAT CAT TGC GAG TCT GGT ATT CA and aaf5_rv: TAA TTT AAG CTG AAG AAT CCA GTC AA) were designed from homologous regions of the annotated AAF/VA gene region from GenBank-deposited pAA plasmid or aaf5A sequences from *E. coli* O111:H21 (accession AB513347, [6]), *E. coli* O127:H21 (accession AB571097), and *E. coli* O6:H^−^ (accession AB571098). PCR reaction conditions were as described above for the *aatA* PCR assay, except that an annealing temperature of 54°C was used.

### *E. coli* Serotyping

Serotyping was performed using antisera against *E. coli* O-antigens 1–181 and *E. coli* H-antigens 1–56 by use of a microtitre agglutination method as described elsewhere [35].

### Determination of *fliC* Genotype

Non-motile (NM or H^−^) strains were analyzed for their flagellar (*fliC*) genotypes by restriction fragment length polymorphism (RFLP) of PCR-products (RFLP-PCR) as described by Prager et al. [35].

### Determination of *stx2* Subtype and Occupation Analysis of Typical Stx-associated Bacteriophage Insertion Sites

The *stx2* subtypes were determined according to Scheutz et al. [36] and occupation of the *wrbA* and *argW* sites for the Stx-associated bacteriophage were tested as described in Shaikh and Tarr [37] and Shringi et al. [38].

### Analysis of Large Plasmids

The determination of plasmid profiles was carried out according to Prager et al. [39].

### Southern Blotting for Identification of pAA Plasmid

Experiments were performed as outlined in Prager et al. using an *aatA* probe [40].

### Analysis of Phylogenetic Type

Phylo-groups A, B1, B2 and D were determined by the multiplex PCR based on the amplification of the genes *chuA*, *yjaA* and the DNA fragment TspE4.C2 according to Clermont et al. [41].

### Analysis of Antibiotic Resistance Profile

Antimicrobial susceptibility of strains was tested against 16 substances by a broth micro dilution method determining the minimum inhibitory concentration according to the guidelines of the European Committee on Antimicrobial Susceptibility Testing (EUCAST) and breakpoints were applied as recommended (www.eucast.com). Antibiotics tested were ampicillin, chloramphenicol, cefoxitin, cefotiam, gentamicin, kanamycin, mezlocillin, mezlocillin/sulbactam, oxytetracyclin, sulfamethoxazol, streptomycin, trimethoprim/sulfamethoxazol, ceftazidim, cefotaxim, ciprofloxacin, and nalidixic acid. *Escherichia coli* ATCC 25922 served as quality control strain.

### Adherence to Hep-2 Cells

A Hep-2 cell adherence assay was performed as previously described [42], [43] with modifications. Briefly, bacteria were grown to exponential growth phase in TSB (Difco), then inoculated 1∶100 in 5 ml TSB containing 1% D-mannose and incubated for 20 h statically at 37°C. Equal growth of the cultures was confirmed by reading OD600. Hep-2 cells, grown to 70 to 90% optical confluence in 24 well plates (in DMEM/10% FCS, GE Healthcare), were washed with PBS and the medium was replaced with DMEM containing 1% D-mannose. Subsequently, 40 ul of the bacterial cultures were added per well. After 3 h of incubation, cells were washed three times with PBS, followed by fixation in ice-cold 70% ethanol on ice for 15 min. Next, samples were stained with Giemsa staining solution (1/20 diluted 0.4% stock solution, diluted in PBS) for 20 min at room temperature. Samples were then rinsed with water, air dried, and mounted for microscopy at 600-fold magnification on a Nikon Eclipse inverted microscope.

### Cytotoxicity Assay

Toxicity towards Vero cells was determined as described previously [44] with modifications. Briefly, strains were grown to exponential phase in TSB (Difco), then diluted 1∶100 in 5 ml TSB, and incubated for 20 h at 37°C with agitation (180 rpm). Next, 100 ul of 8-fold to 512-fold DMEM (GE Healthcare) diluted cell free culture supernatants of the TSB-grown strains were added to washed confluent Vero cell monolayers in 100 ul DMEM/10% FCS in 96 well plates in triplicates. For each experiment fresh culture supernatants were produced and equal growth of the bacterial cultures was confirmed by OD600 readings. After 48 h of incubation at 37°C, supernatants were analyzed for LDH release by means of the CytoTox96 Non-Radioactive Cytotoxicity Assay (Promega) according to the manufacturer’s protocol. All values shown are corrected by the background reading of the diluted culture supernatants. In all assays, 256-fold diluted *E. coli* culture supernatants were determined as best discriminative and are therefore shown.

### Macrorestriction Analysis/Pulsed-field Gel Electrophoresis

The macrorestriction analysis by pulsed-field gel electrophoresis (PFGE) using the restriction enzyme XbaI was carried out as specified by Prager et al [45].

### Multi Locus Sequence Typing

The multiple locus sequence typing (MLST) was performed as described in Prager et al [45]. The MLST alleles and sequence types (ST) were assigned in concordance with the *E. coli* MLST database at http://mlst.ucc.ie/mlst/mlst/dbs/Ecoli/ [46].

## Results

### Identification of Two EHEC Strains with Additional EAEC Marker Genes from Human Infections

**Figure 1:**
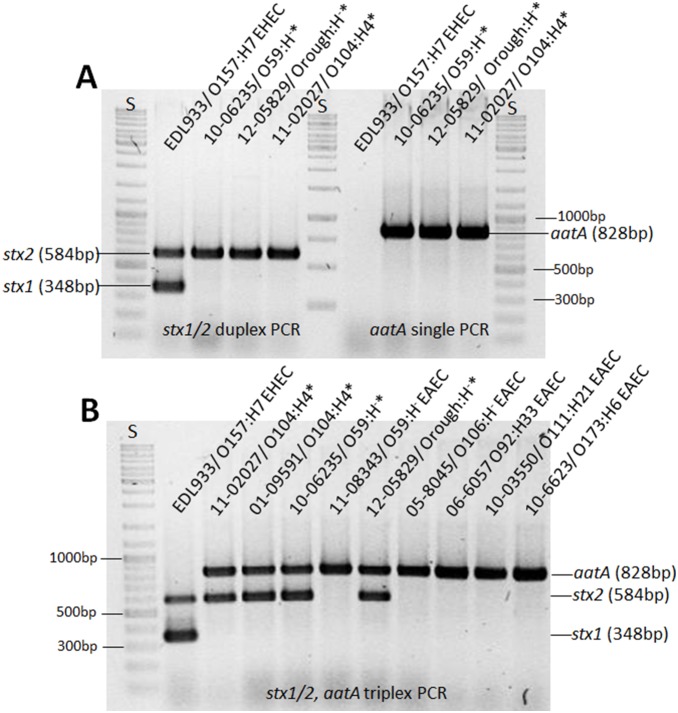
Identification of two novel EHEC/EAEC strains. (**A**) Duplex *stx1/2* PCR analysis (left side of panel) and single *aatA* PCR analysis (right side of panel) of EHEC EDL933, known EHEC/EAEC 11-02027 (O104:H4 outbreak 2011), and the two novel EHEC/EAEC: 10-06235 (O59:H^−^) and 12-05829 (Orough:H^−^). (**B**) Novel Triplex PCR analysis for simultaneous detection of *stx1/2* and *aatA* in different EHEC, EHEC/EAEC, and EAEC strains. S = bp standard; *EHEC/EAEC.

After the large outbreak caused by EHEC/EAEC O104:H4 in 2011, we analyzed whether additional EHEC/EAEC were among the strains deposited in the EHEC collection of the German National Reference Centre for *Salmonella* and other Bacterial Enteric Pathogens (NRC). Between 2008 and 2012 4059 EHEC samples from mainly human origin were analyzed at NRC. After exclusion of O104:H4 EHEC/EAEC, 2435 strains were investigated for the presence of *stx1, stx2, eaeA*, and *ehxA* genes. 268 *eaeA*- and *ehxA*-negative EHEC strains of O-serotypes commonly associated with EAEC, rare EHEC O-serotypes, and untypable EHEC strains were chosen for further analysis of EAEC marker genes. As a first step in our study, analysis of the diagnostic EAEC marker gene *aatA* was employed [47]. We detected two strains, one from 2010 (strain 10-06235) and another from 2012 (strain 12–05829), showing a positive PCR amplification for *stx2* and *aatA* and therefore represent novel EHEC/EAEC hybrids (Fig. 1A). To facilitate future analysis of EHEC/EAEC hybrid strains, we designed a primer pair for detection of *aatA* which can be combined with the earlier published primers for the *stx1/2* duplex PCR in a novel triplex PCR approach [31]. We tested known EHEC/EAEC and a variety of classical EAEC for correct determination of *aatA* presence (Tab. 1). As shown in Fig. 1B, all of the tested EAEC or EHEC/EAEC strains yielded an *aatA* amplificate (Fig. 1B). Further presence of *stx1* and/or *stx2* was correctly detected in the here investigated EHEC or EHEC/EAEC strains (Fig. 1B). In summary, our data show that EHEC/EAEC strains are infrequently associated with human disease in Germany. Further, we found two novel potential EHEC/EAEC strains and we developed a triplex PCR to facilitate future detection of EHEC/EAEC.

### Strain 10-06235 Belongs to the Rare Serovar O59:H^−^ and to Rare ST1136, and Represents a Novel EHEC/EAEC with Type IV Aggregative Fimbriae

**Figure 2:**
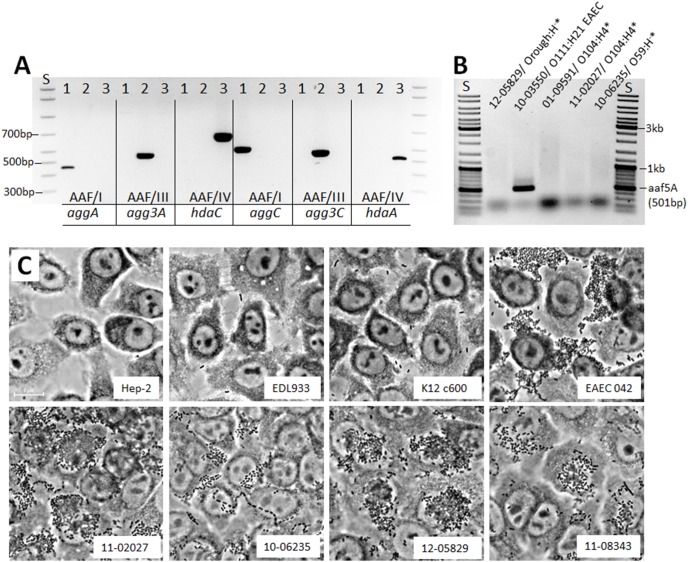
The novel O59:H^−^ EHEC/EAEC codes for AAF/IV fimbriae and both novel EHEC/EAEC show aggregative adherence. (**A**) PCR-based determination of AAF type I, III, and IV genes of the EHEC/EAEC O59:H^−^ strain (3) compared to two known EHEC/EAEC O104:H4 (1 - outbreak 2011 and 2 - HUSEC 041) strains. (**B**) PCR-based determination of AAF/V-coding gene *aaf5A* of different EHEC/EAEC strains and an O111:H21 EAEC strain as positive control. (**C**) Aggregative adherence of the two novel EHEC/EAEC strains to Hep-2 cells. The controls EHEC EDL933 (O157:H7) and *E. coli* K12c600 without aggregative adherence and the aggregative adherence reference strains EAEC 042 (O44:H18), EHEC/EAEC 11-02027 (O104:H4 outbreak 2011) were analyzed in addition to the EHEC/EAEC 10-06235 (O59:H^−^), EHEC/EAEC 12-05829 (Orough:H^−^), and the EAEC 11-08343 (O59:H^−^) strains. Images were taken at 600-fold magnification. S = bp standard; *EHEC/EAEC.

To characterize the novel EHEC/EAEC candidates, we first analyzed the strain from 2010 (strain 10-06235) which was isolated from a patient with bloody diarrhea. It belonged to the serovar O59:H^−^ and to ST1136 [46]. In the *E. coli* MLST database at http://mlst.ucc.ie/mlst/mlst/dbs/Ecoli/only one strain of unknown serotype isolated from a patient with urinary tract infection in Rio de Janeiro/Brasil in 2006 (strain HMMC322, source_lab Beatriz M. Moreiro) has been assigned to this sequence type. Among the intestinal pathogenic *E. coli* strains within the strain collection of NRC, only one other strain of serovar O59:H^−^ (strain 11-08343) was found between 1993 and 2012 demonstrating the rare occurrence of this serovar in human infections in Germany. This strain, isolated from a patient with bloody diarrhea in 2011, was a classical EAEC strain and did not possess an *stx* gene (Fig. 1B). Both O59:H^−^ strains showed a flagellar *fliC* genotype *fliC_H19_* (data not shown) and the presence of type IV aggregative AAF fimbriae as determined by amplification of the *hdaA* and *hdaC* genes (Fig. 2A and data not shown). In contrast, the *aggA*, *aggC* or *agg3A* and *agg3C* genes, representing the genes for type I or type III AAF found in the 2011 O104:H4 outbreak strain or in HUSEC041, another EHEC/EAEC of serovar O104:H4 isolated from a HUS patient in 2001, respectively, were not detected (Fig. 2A) [48]. The genes corresponding to type II or type V AAF were not amplified in the O59:H^−^ strains (data not shown and Fig. 2B). As expected from the detection of AAF genes, the O59:H^−^ as well as the O104:H4 strains showed the characteristic aggregative adherence to Hep-2 cells (Fig. 2C). We therefore conclude that the O59:H^−^ strain from 2010 indeed represents a novel EHEC/EAEC belonging to a rare serovar and ST.

### The Novel O59:H^−^ EHEC/EAEC Strain Possesses a Shiga Toxin Gene of the Subtype *stx2a* and Further Genes Characteristic for EHEC and EAEC

**Figure 3:**
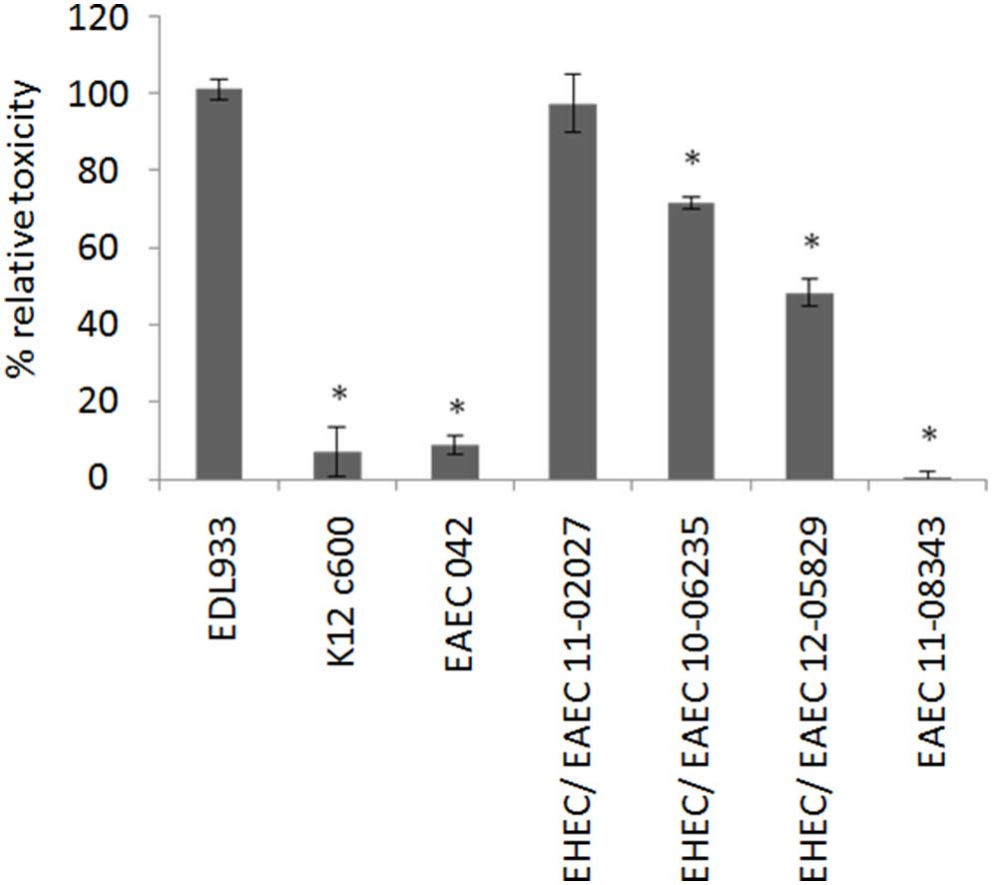
Toxicity of the two novel EHEC/EAEC strains towards Vero cells. As a positive control EHEC EDL933 and as negative controls *E. coli* K12c600 and EAEC 042 were incubated with Vero cells. Further EHEC/EAEC 11-02027 (O104:H4 outbreak 2011), EHEC/EAEC 10-06235 (O59:H^−^), EHEC/EAEC 12-05829 (Orough:H^−^), and EAEC 11-08343 (O59:H^−^) were analyzed. Toxicity of EHEC EDL933 served as a quantitative reference and was set to 100%. Bars represent means and standard deviations of triplicate samples and 256-fold diluted culture preparations. Asterisks indicate significantly lower cytotoxicity compared to the EDL933 reference strain, however all EHEC/EAEC strains were significantly more cytotoxic then the *E. coli* K12 control, the EAEC 042, as well as EAEC 11-08343 (O59:H^−^) strains (two tailed student’s t-test type 1, p<0.01; p<0.03 for EHEC/EAEC 12-05829 compared to *E. coli* K12).

Next, we analyzed the Shiga toxin gene type associated with the novel EHEC/EAEC and found that the O59:H^−^ strain exhibited the subtype *stx2a* [36]. PCR analysis revealed occupation of the *argW* but not the *wrbA* site suggesting integration of the Stx-associated bacteriophage into the *argW* site (data not shown) [38]. The *stx2AB* gene sequence of the O59:H^−^ strain (GenBank accession: KJ158456) differed only by two nucleotides at position 129 (A/G) and 867 (T/C) in *stx2A* from the sequence of the 2011 O104:H4 outbreak strain (GenBank accession: CP003289). The StxA and StxB protein sequences of both strains were identical. We compared the toxicity of Stx released by the novel EHEC/EAEC to that of the 2011 O104:H4 outbreak strain and the classical EHEC EDL933. Although the toxicity of the new EHEC/EAEC strains towards Vero cells was lower than that of the prototypic EHEC O157:H7 strain EDL933, it was in each of the novel EHEC/EAEC strains significantly higher than the toxicity of Stx-negative control strains; indicating that the hybrid EHEC/EAEC strains produced biologically active Stx2 (Fig. 3). We further analyzed additional marker genes of classical EHEC and EAEC by means of PCR [4]. The following genes frequently associated with EHEC were detected for both of the O59:H^−^ strains: *iha* (gene for IrgA adhesion homolog), *lpfA_O26_* (gene for structural subunit of long polar fimbriae (LPF) of EHEC O26), *lpfA_O113_* (gene for structural subunit of LPF of EHEC O113), and *irp2* (gene for a component of iron uptake system on high pathogenicity island) (Tab. 2). In addition to the already mentioned AAF genes *hdaA* and *hdaC* (Fig. 2A), the presence of the following EAEC markers was determined in both O59:H^−^ strains: *aggR* (transcriptional regulator gene), *aap* (dispersin gene), *set1a* (*Shigella* enterotoxin subunit A gene), *set1b (Shigella* enterotoxin subunit B gene), *pic* (coding for Pic, a protein involved in intestinal colonization), *sigA* (gene for the SPATE protein, i.e. the cytotoxic serin protease autotransporter of Enterobacteriaceae, [49]) and *iucA* (gene for the siderophore aerobactin). *astA,* the gene for EAEC heat stable enterotoxin 1, EAEST1, was not detected in these strains (Tab. 2).

**Table 2:** Characteristics of the novel EHEC/EAEC strains analyzed in the study compared to the O59:H^−^ EAEC and the O104:H4 2011 outbreak strain.

				genes frequently associated with EHEC						genes frequently associated with EAEC								
Pathovar/notes	Isolate number	Serovar	AAF type	*stx*	*iha*	*lpfA*_O26_	*lpfA*_O113_	*irp*2	*terA*	*aatA*	*aap*	*set1a*	*set1b*	*pic*	*sigA*	*iucA*	*astA*	*aggR*
**Novel EHEC/EAEC,** **this study**	**10-06235**	**O59:H^−^ (** ***fliC_H19_*** **)**	*hdaA/C*(type IV)	2a	+	+	+	+	**−**	+	+	+	+	+	+	+	**−**	+
EAEC, this study	11-08343	O59:H^−^ (*fliC_H19_*)	*hdaA/C*(type IV)	**−**	+	+	+	+	**−**	+	+	+	+	+	+	+	**−**	+
**Novel EHEC/EAEC,** **this study**	**12-05829**	**Orough:H^−^ (** ***fliC_H8_*** **)**	unknown	2b	**−**	+	+	+	*+*	+	**−**	**−**	**−**	**−**	**−**	**−**	+	**−**
EHEC/EAECoutbreak 2011Germany [7]	11-02027	O104:H4	*aggA/C*(type I)	2a	+	+	+	+	+	+	+	+	+	+	+	+	**−**	+

### The Novel O59:H^−^ EHEC/EAEC Strain Possesses Three Large Plasmids and Resistance towards Three Antibiotic Classes

**Figure 4:**
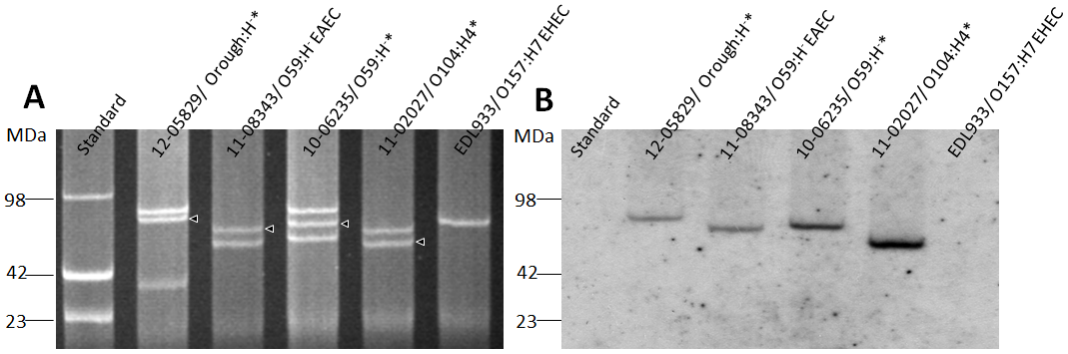
Plasmid profile of the two novel EHEC/EAEC strains and identification of the pAA plasmid. (**A**) EHEC/EAEC 12-05829 (Orough:H^−^), EAEC 11-08343 (O59:H^−^), EHEC/EAEC 10-06235 (O59:H^−^), EHEC/EAEC 11-02027 (O104:H4 outbreak 2011), and EHEC EDL933 (O157:H7) were analyzed for their plasmid profile. *E. coli* reference strain 39R861 plasmids served as molecular mass standard. (**B**) Southern hybridization with plasmid DNA of the same strains as mentioned in (A) was performed using a digoxigenin-labelled *aatA* gene probe. *EHEC/EAEC; ?pAA plasmid.

The novel O59:H^−^ EHEC/EAEC strain was analyzed for the presence of plasmids and three large plasmids of ∼50, ∼60, ∼70 MDa were detected (Fig. 4A). The classical O59:H^−^ EAEC strain possessed two large plasmids of ∼50 and ∼60 MDa (Fig. 4A) and the 2011 O104:H4 outbreak strain two large plasmids of ∼55 and ∼60 MDa. Southern blot analysis using an *aatA* probe identified in both O59:H^−^ strains the ∼60 MDa plasmid (i.e. the second largest plasmid in the EHEC/EAEC strains and the larger plasmid in the EAEC strain) as the pAA plasmid (Fig. 4B). Having in mind the unusual multidrug resistance of the O104:H4 outbreak strain from 2011 towards a variety of antibiotics, including sulfonamides, streptomycin, trimethoprim/sulfonamide, ampicillin, 3^rd^ generation cephalosporines, and others [4], [5], [7], we also analyzed the O59:H^−^ strains for their resistance phenotype. In the case of the novel O59:H^−^ EHEC/EAEC, we found resistance towards sulfonamides, streptomycin, and trimethoprim/sulfonamide. The O59:H^−^ EAEC was susceptible to all antibiotics investigated. Clearly, the 2011 outbreak strain showed resistance towards more (at least seven) substance classes than the O59:H^−^ strains which were resistant towards three substance classes or completely susceptible (Tab. 1).

### PFGE Analysis Shows that the O59:H^−^ EHEC/EAEC Isolate is not Closely Related to the O59:H^−^ EAEC Isolate

**Figure 5:**
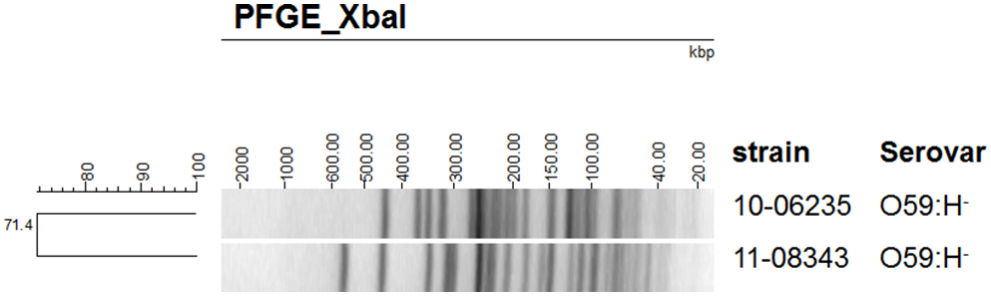
Pulsed-field gel analysis after XbaI macrorestriction shows that the EHEC/EAEC O59:H^−^ (upper lane) and EAEC O59:H^−^ (lower lane) are not closely related.

PFGE analysis was used to determine the degree of genetic relatedness between the two O59:H^−^ strains. As already suggested by the presence or absence of *stx2*, the different plasmid profiles, and antibiotic resistance phenotypes, the EHEC/EAEC and the EAEC were not closely related. PFGE analysis showed differing restriction patterns for both strains with about 71% similarity and therefore corroborated our earlier findings (Fig. 5).

### Strain 12-05829 was Typed as Orough:H^−^, Belongs to ST26, and Shows an Aggregative Phenotype

We next analyzed the strain from 2012 (strain 12-05829) which comprised the *stx2* and *aatA* genes (Fig. 1A). The strain was isolated from a patient with diarrhea. It could not be attributed to any known serovar due to its rough character and lacking motility. The strain showed a flagellar *fliC* genotype *fliC_H8_* (data not shown). ST26 was assigned [46] and according to the MLST database, only two *E. coli* strains of the serovar O40:H8, one from a human without disease symptoms (Wunsiedel/Germany, strain 6840/96, source_lab H. Karch) and another from a patient with diarrhea (Wuerzburg/Germany, strain 3199/98, source_lab H. Karch), have been found associated with this sequence type and no clonal complex has been defined yet. Within the strain collection of NRC, 11 other EHEC strains of the serovar O40:H8 have been found. All were negative for *aatA* as analyzed by PCR (data not shown). For the novel *aatA*-positive Orough:H^−^ strain from 2012 not any of the known type I–V AAF-encoding genes was detected but aggregative adherence to Hep-2 cells was found (data not shown and Fig. 2B and C). Therefore, we conclude that this novel EHEC/EAEC isolate likely possesses so far unclassified AAF [25], [34]. The strain harbored an *stx2* gene of type *stx2b*, showed toxicity towards Vero cells (Fig. 3), and three large plasmids of ∼40, ∼75 and ∼80 MDa (Fig. 4A and data not shown). Southern Blot analysis of its plasmid profile indicated that the ∼75 MDa DNA represents the pAA plasmid (Fig. 4B). Strain 12-05829 was susceptible to all of the antibiotics used in the study (Tab. 1).

We analyzed strain 12-05829 for additional marker genes of classical EHEC and EAEC [4]. The following genes associated with EHEC were detected: *lpfA_O26_* (gene for structural subunit of long polar fimbriae (LPF) of EHEC O26), *lpfA_O113_* (gene for structural subunit of LPF of EHEC O113), *terA* (gene of tellurite resistance gene cluster), and *irp2* (gene of component of iron uptake system on high pathogenicity island) (Tab. 2). Further, the presence of *astA* (gene for EAEC heat stable enterotoxin 1 EAEST1), an EAEC marker, was determined. The EAEC markers *aggR* (transcriptional regulator gene) and *aap* (dispersin gene) as well as some others were not detected (Tab. 2).

## Discussion and Conclusion

We here identified and characterized two novel EHEC/EAEC hybrid strains which were isolated from humans in Germany in 2010 and in 2012. Since the large outbreak due to EHEC/EAEC O104:H4 2011 in Germany, attention was drawn on these mixed *E. coli* pathovars and so far such strains have been seldom described. Nevertheless, at least eight other O104:H4 EHEC/EAEC strains have been documented, for example a strain designated HUSEC041 causing HUS in Germany in 2001, two French strains from 2004 and 2009, and five additional strains causing HUS in France in 2011. Those strains clearly differ from the 2011 outbreak strain, for example in their PFGE macrorestriction profile, AAF type, antibiotic resistance, plasmid profile, virulence gene sets, or SNPs on the genomic level [9], [16], [50]–[52]. The relatively frequent occurrence of EHEC/EAEC among the serovar O104:H4 strains suggests that either certain EAEC serovars/strains might be more susceptible to acquire EHEC determinants, such as an *stx*-harboring phage, or that there are certain EHEC/EAEC ancestors which successfully adapted to survive specific selection conditions.

Although it has been shown that the *stx1* phage is easily transferred to *E. coli* K-12 in the intestinal tract of mice [53], just a few other *E. coli* serovars are reported to reveal strains with EHEC/EAEC features. An O111:H2 strain causing HUS in France in 1996 exhibits *stx2*, AAF and was *eaeA* and *ehxA* negative and the first described EHEC/EAEC [10], [11], [54]. Further, an O86:H^−^ strain with *stx2* and AAF marker genes but no *eaeA* was isolated from a pediatric patient with HUS and bloody diarrhea in Japan in 1999 [55]. An O111:H21 strain associated with a household outbreak in Northern Ireland in 2011 including a HUS case in a child was characterized on the genomic level. It was *eaeA* negative and it belongs to ST40, a sequence type comprising a diversity of *E. coli* pathovars, such as STEC, EAEC, EPEC, and non-pathogenic *E. coli* [6]. The strain possesses *stx2c* and a recently described type of AAF, designated type V AAF [6] (see also below). Of note, hybrid *E. coli* pathovars showing EHEC properties combined with features of yet other pathotypes were recently reported [56], [57].

In our study, we found two novel EHEC/EAEC strains associated with human disease; one of those belonged to the rare serovar O59:H^−^ (flagellar genotype *fliCH19*) and the rare MLST ST1136. The other strain was a non-motile strain with rough LPS belonging to the rare MLST ST26. Since two other strains of ST26 were found in the MLST database which were serotyped as O40:H8, we tested the Orough:H^−^ strain for genetic elements coding for the surface antigens related to those in the O40:H8 strains. Accordingly, the *fliC* genotype was *fliCH8*. Screening the literature on *E. coli* of O-serotype O59 or O40 revealed only a few publications addressing the characterization of the antigenic polysaccharides or using the strain as a reference strain to evaluate survival of *E. coli* [58]–[61]. Our finding therefore confirms that EHEC/EAEC in the so far known cases belong to *E. coli* serovars rarely described to cause humans disease.

EHEC/EAEC strains show a high virulence potential. This is illustrated by the O104:H4 outbreak in 2011 where an extraordinarily high number, more than 20%, of the registered patients, developed the severe pathology HUS [7], [3]. In the case of classical EHEC, the proteins coded by the LEE locus and most importantly the adhesin EaeA promote intimate attachment to the host cells and therefore facilitate Stx application and action [1]. The so far described EHEC/EAEC strains do not possess *eaeA* and it is argued that attachment driven by AAF may be at least as effective as that mediated by EaeA. The ability of EHEC/EAEC strains to form adhesive aggregates may even increase Stx quantities at a specific site and therefore the toxic capacity of the bacteria [8], [10], [14], [15]. This notion is underlined by an observation involving the 2011 O104:H4 strain. Studies determined that the pAA plasmid can be lost in the human gut and the authors hypothesized that loss of the adhesive aggregative properties attenuates virulence, a point which might have contributed to the limited number of secondary infections [14].

So far five different types of AAF have been assigned to specific gene components coded on the pAA plasmid [6], [20]–[23]. In a study which analyzed 17 EAEC isolates from Danish diarrhea patients, type I AAF turned out to be the most common AAF type found in 9 of the isolates, an AAF type also present in the 2011 outbreak strain [4], [7], [20]. Further, Boisen et al. described that 8 strains could not be assigned to other known AAF types II and III although showing aggregative adherence, suggesting that so far unknown types of AAF do exist. Indeed, for five strains, it was found that the pilin gene cluster showed several important differences from the other AAF types which was the reason to classify them into AAF/IV [20]. The recently described O111:H21 EHEC/EAEC strain was classified as an AAF/V-harboring strain [6]. However, it was not described how the novel AAF type discriminates from the ones previously assigned and how this type may be detected by means of PCR. AAF/V so far represented by the major pilin subunit gene *aaf5a* of EHEC/EAEC O111:H21 [6], *E. coli* O127:H21 (GenBank accession AB571097), and *E. coli* O6:H^−^ (GenBank accession AB571098) showed no homology to the major pilin subunit gene of AAF/I to AAF/IV on the DNA level. About 30% homology to related proteins of AAF/III was found on the protein level and similarities were especially present in the N-terminal regions of the proteins. Therefore, we have designed a PCR assay amplifying the *aaf5A* gene (Fig. 2B). No type I–V AAF fimbriae could be assigned to the novel Orough:H^−^ EHEC/EAEC strain by the here used PCR-based AAF gene detection. Therefore, other AA fimbrial subunits such as those not yet known or completely distinct aggregation factors may be present in this strain, as suggested by Harrington et al. [25]. Future experiments involving genome or pAA sequence analysis may give an insight into the nature of the aggregation determinants.

Due to the high virulence potential of EHEC/EAEC as outlined above, it is necessary to keep track of EHEC/EAEC causing human disease and to characterize their virulence determinants. To facilitate recognition of EHEC/EAEC strains in the future, we developed a triplex PCR assay for concomitant detection of *stx1, stx2*, and *aatA*. This assay extends on the duplex *stx* PCR assay previously described to amplify both *stx1* and *stx2* [31] by adding a primer pair for *aatA* detection (Fig. 1B).

In conclusion, we described two novel EHEC/EAEC strains isolated from human disease cases in Germany in 2010 and 2012; one of those originating from a bloody diarrhea. Those strains belong to MLST sequence types and/or serotypes seldom associated with human disease and in addition to *stx2* harbor EAEC characteristics which qualify them to cause severe disease.
